# *Aspergillus flavus* infection triggered immune responses and host-pathogen cross-talks in groundnut during *in-vitro* seed colonization

**DOI:** 10.1038/s41598-017-09260-8

**Published:** 2017-08-29

**Authors:** Spurthi N Nayak, Gaurav Agarwal, Manish K Pandey, Hari K Sudini, Ashwin S Jayale, Shilp Purohit, Aarthi Desai, Liyun Wan, Baozhu Guo, Boshou Liao, Rajeev K Varshney

**Affiliations:** 1 International Crops Research Institute for the Semi-Arid Tropics (ICRISAT), Hyderabad, India; 2 Crop Protection and Management Research Unit, USDA-Agricultural Research Service, Tifton, GA USA; 3 University of Georgia, Department of Plant Pathology, Tifton, GA USA; 4 Oil Crops Research Institute (OCRI), Chinese Academy of Agricultural Sciences (CAAS), Wuhan, China; 5 The University of Western Australia, Crawley, WA Australia; 6 Department of Biotechnology, Present Address: University of Agricultural Sciences, Dharwad, India

## Abstract

Aflatoxin contamination, caused by fungal pathogen *Aspergillus flavus*, is a major quality and health problem delimiting the trade and consumption of groundnut (*Arachis hypogaea* L.) worldwide. RNA-seq approach was deployed to understand the host-pathogen interaction by identifying differentially expressed genes (DEGs) for resistance to *in-vitro* seed colonization (IVSC) at four critical stages after inoculation in J 11 (resistant) and JL 24 (susceptible) genotypes of groundnut. About 1,344.04 million sequencing reads have been generated from sixteen libraries representing four stages in control and infected conditions. About 64% and 67% of quality filtered reads (1,148.09 million) were mapped onto A (*A. duranensis*) and B (*A. ipaёnsis*) subgenomes of groundnut respectively. About 101 million unaligned reads each from J 11 and JL 24 were used to map onto *A. flavus* genome. As a result, 4,445 DEGs including defense-related genes like senescence-associated proteins, resveratrol synthase, 9s-lipoxygenase, pathogenesis-related proteins were identified. In *A. flavus*, about 578 DEGs coding for growth and development of fungus, aflatoxin biosynthesis, binding, transport, and signaling were identified in compatible interaction. Besides identifying candidate genes for IVSC resistance in groundnut, the study identified the genes involved in host-pathogen cross-talks and markers that can be used in breeding resistant varieties.

## Introduction

Aflatoxins are a group of carcinogenic mycotoxins produced mainly by *Aspergillus flavus* and *A. parasiticus*. They are considered to be a threat to human health, global food safety, and security^1, 2^. Maize (*Zea mays* L.) and groundnut (*Arachis hypogaea* L.) are the most susceptible crops to aflatoxin contamination and serve as the main source of aflatoxin exposure for humans. The aflatoxins were reported as growth retardants in children^3^ and immune-suppressors leading to AIDS^4, 5^. Aflatoxin B1 produced by *A. flavus* and *A. parasiticus* in groundnut is considered to be the major cause of liver cancer along with the chronic infection with hepatitis B and C viruses and acute aflatoxicosis^6^.

Groundnuts tend to be colonized and contaminated by *Aspergillus* spp. at pre-harvest, during harvest, post-harvest drying, in storage and also during transport covering the whole value chain^7, 8^. Use of cultivars resistant to seed invasion by *Aspergillus* spp. is one of the possible means of reducing aflatoxin contamination during groundnut storage with no extra input cost for the farmers. Development of aflatoxin resistant groundnut varieties has been a challenging task for breeders due to lack of availability of reliable resistance sources, poor understanding of plant-fungus interactions and large environmental influence. Three types of resistance mechanisms namely *in-vitro* seed colonization resistance (IVSC), resistance to pre-harvest aflatoxin contamination (PAC) and resistance to aflatoxin production in seeds have been reported in groundnut. However, the aflatoxin is produced only in the cotyledons of groundnut kernels after fungal infection^9^. Sources of resistance to these mechanisms were identified independently^7, 10, 11^. Therefore, there is a need to understand the molecular mechanisms of resistance to aflatoxin contamination.

The differential transcriptome analysis based on next-generation sequencing (NGS), can provide insights to understand resistance mechanisms and offers the possibilities of identifying novel genes and splice variants^12, 13^. Few reports on expression profiles are available for selected tissues of plant growth and development in groundnut using either microarray^14–16^ or transcriptome sequencing^17–22^. Transcriptome studies have been carried out for biotic stresses like late leaf spot^23, 24^ and bacterial wilt^25^. In addition, transcriptome studies related to *Aspergillus* spp. infection^26, 27^ under drought conditions^28^ have also been carried out using microarray technology. Very recently, transcript profiling of resistant and susceptible groundnut post-harvest seeds has been undertaken in response *A. flavus* infection^29^. However, none of the above studies related to aflatoxin contamination provided the decisive insights on genomic control and solution for their deployment in developing improved varieties.

As aflatoxin is produced preferentially in the seed and IVSC is the first step towards fungal infection, it is considered as the most important stage during aflatoxin contamination. Identifying molecular mechanisms and candidate genes associated with IVSC resistance could lead to a breakthrough in controlling fungal colonization and aflatoxin contamination in groundnut. Furthermore, the availability of genome sequence information of diploid progenitors of tetraploid cultivated groundnut^30, 31^ and advances in next generation sequencing (NGS) technologies provide tools to understand the genomic control for such complex traits^32, 33^. The present study, therefore, aims identification of candidate genes and enhancing understanding of resistance mechanisms for IVSC using RNA-seq approach in groundnut.

## Results

### Microscopic observation and aflatoxin estimation

**Figure 1:**
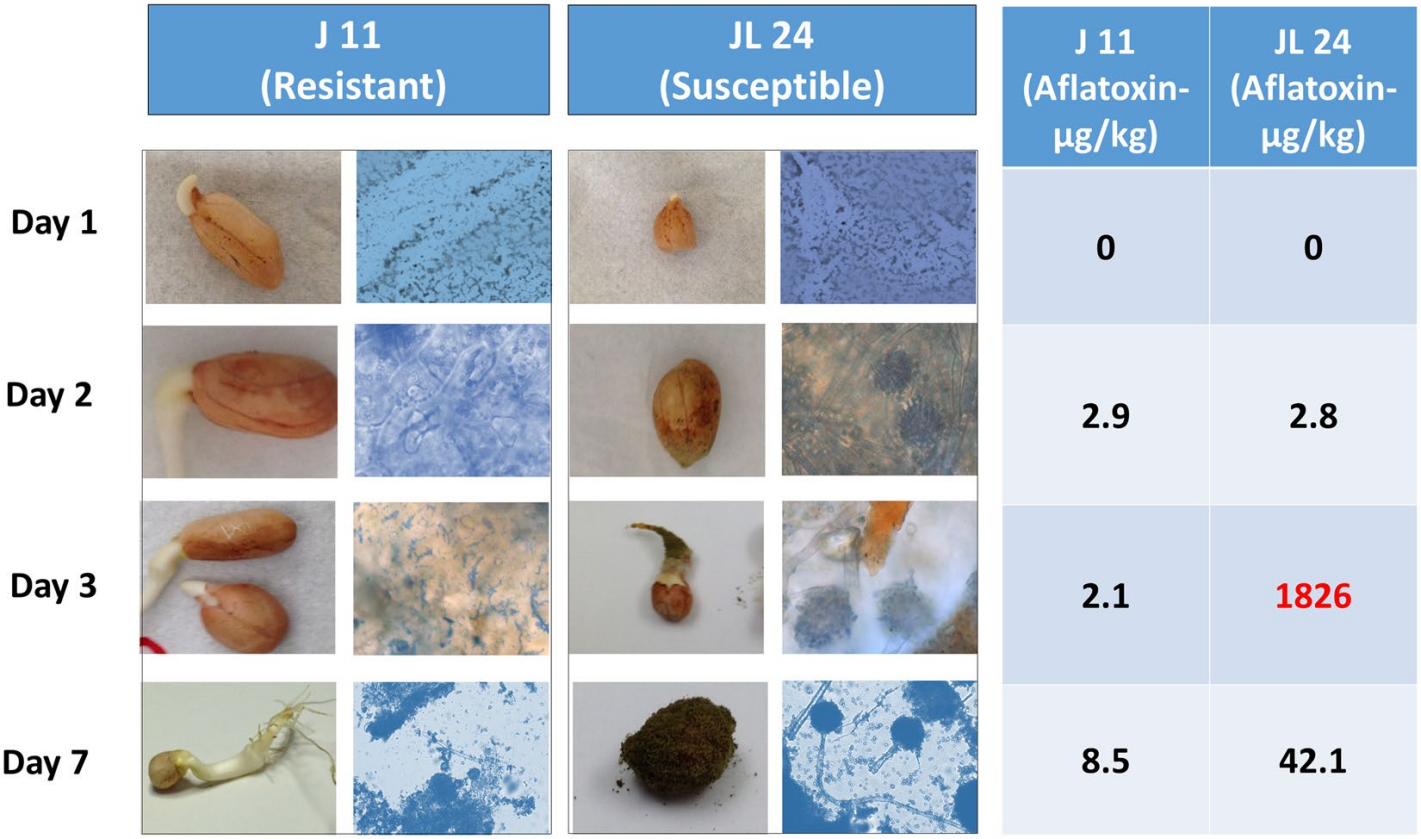
Phenotypic observations for seeds of J 11 and JL 24 during *in-vitro* seed colonization by *Aspergillus flavus* at different time points along with microscopic observation and aflatoxin estimation. The figure shows the microscopic observations of seed coat after fungal inoculation in J 11 (resistant) and JL 24 (susceptible) that clearly shows the presence of mycelium at Day 3 and sporulation at Day 7 after inoculation.

Observation of the seed coat of the control and infected seeds using the florescence microscope showed considerably low mycelial growth at first day after inoculation (1DAI) in both the genotypes (J 11 and JL 24). The infected samples of JL 24 began to show higher mycelial growth than that of J 11 at 2DAI and even more at 3DAI. Sporulation was observed in JL 24 at 7DAI as compared to no or very less number of spores in J 11. There was a clear inhibition of fungal colonization on groundnut seeds in J 11 compared to JL 24. In control conditions, both the genotypes showed uniform germination while after infection, JL 24 failed to germinate due to heavy fungal invasion and colonization. Highest toxin production (1,826 µg aflatoxin /kg seed) was observed at 3DAI in the case of JL 24 (Fig. 1).

### Transcriptome sequencing and gene expression analysis

The paired-end (2 × 100 bp) sequencing of sixteen samples generated 268.81 Gb of data comprising of 1,344.04 million raw reads with an average of 84 million reads per sample (Table 1) and was submitted to National Centre for Biotechnology Information (NCBI) with BioProject ID PRJNA355201 and Sequence Read Archive SRP094387. After the stringent quality check, a total of 1,064.21 million reads along with the high quality (QC ≥ 30) 83.88 million singleton were used for mapping onto A (*A. duranensis*) and B (*A. ipaёnsis*) subgenomes of groundnut. Percent mapping was slightly higher on the B subgenome (770.84 million reads; 67.14% of quality filtered) compared to the A subgenome (737.75 million reads; 64.26% of quality filtered) (Table 1). Interestingly, about 74–77% of the reads were mapped to exonic region, 7% to intronic region and about 11% to inter-genic regions of both A and B subgenomes (Supplementary Table 1). A total of 19,569 and 20,299 genes expressed during infection could be mapped to A and B subgenomes, respectively. Out of which 19,398 and 20,227 genes were mapped on the pseudomolecules of A and B subgenomes, rest of 171 and 72 genes were mapped onto scaffolds, hence do not have assigned coordinate information.

**Table 1:** Summary of sequencing data generated and mapped on A and B subgenomes of groundnut.

Library information	Total reads (millions)	High quality reads (millions, >Q30 filtered)	High quality singletons (millions, >Q30 filtered)	Number of reads (millions) uniquely mapped on A subgenome	Number of reads (millions) uniquely mapped on B subgenome
J 11_Control_1DAI	100.20	79.69	6.02	63.17	63.50
J 11_Control_2DAI	90.06	71.10	5.59	52.88	53.06
J 11_Control_3DAI	88.01	69.53	5.45	51.97	52.19
J 11_Control_7DAI	90.68	67.66	6.76	42.72	47.02
J 11_Infected_1DAI	73.82	57.92	4.69	43.00	44.37
J 11_Infected_2DAI	77.79	56.41	6.35	28.15	32.68
J 11_Infected_3DAI	75.07	60.19	4.49	41.01	42.59
J 11_Infected_7DAI	75.02	59.07	4.80	38.00	40.55
JL 24_Control_1DAI	85.28	72.09	5.36	51.74	54.27
JL 24_Control_2DAI	89.96	73.30	4.92	55.72	56.53
JL 24_Control_3DAI	89.73	70.04	4.99	50.50	51.52
JL 24_Control_7DAI	86.77	67.54	5.35	43.72	47.48
JL 24_Infected_1DAI	85.61	67.05	5.59	44.12	47.64
JL 24_Infected_2DAI	89.88	75.60	4.77	47.06	51.05
JL 24_Infected_3DAI	69.52	56.68	3.86	43.47	43.42
JL 24_Infected_7DAI	76.62	60.33	4.89	40.53	42.96
Total	1344.04	1064.21	83.88	737.75	770.84

The unaligned reads i.e. 101.4 and 101.3 million reads from J 11 and JL 24 genotypes were assembled and used to map onto *Aspergillus* genome. An average of 4.45% and 5% of reads of infected samples of J 11 and JL 24 genotypes were mapped onto the fungal genome. The study indicated marginally higher mapping in JL 24, as compared to J 11 genotype. As expected the reads from control samples did not have any alignment on fungal genome indicating the sanity of the experiment.

### Differentially expressed genes (DEGs) during *Aspergillus* infection

**Figure 2:**
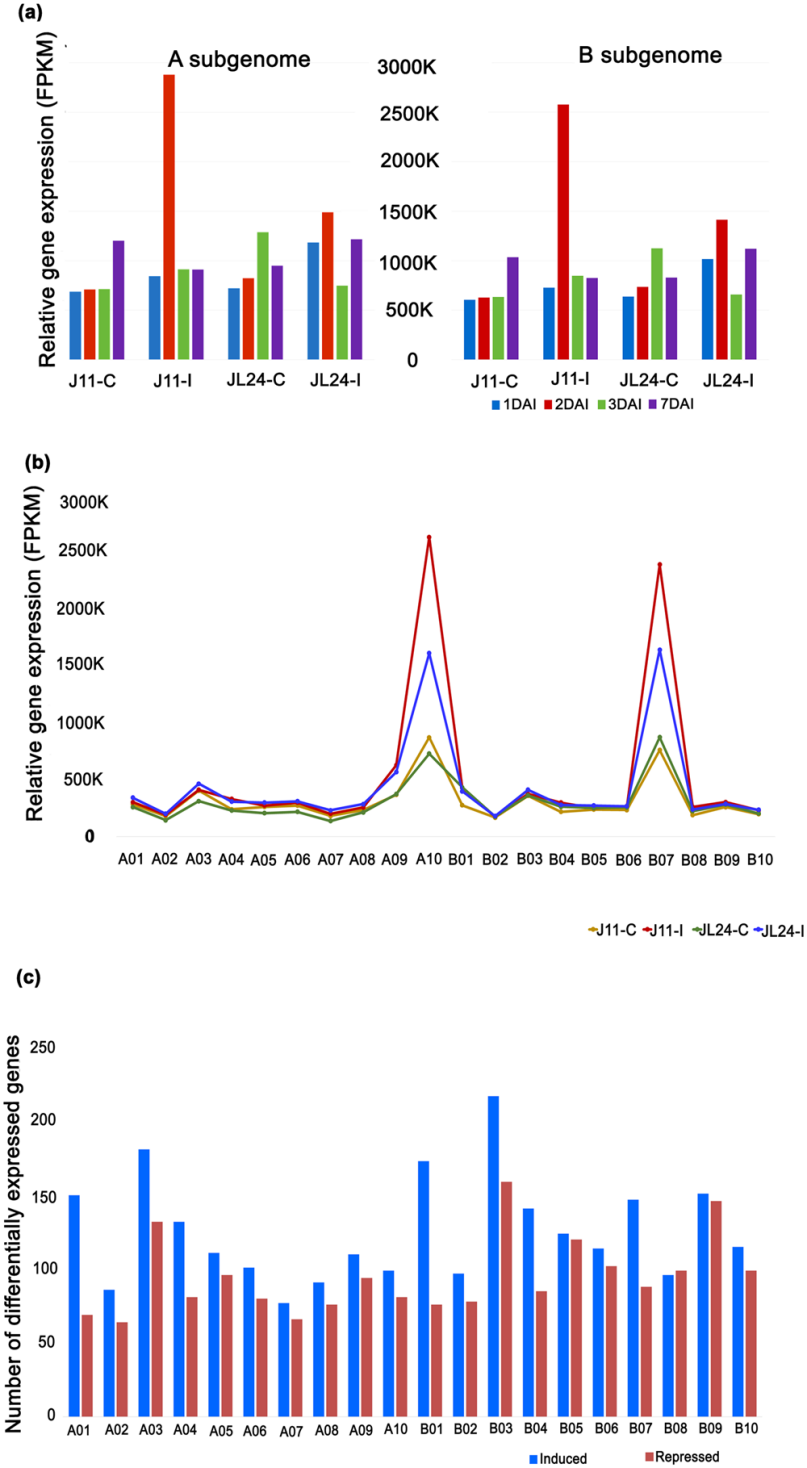
Gene expression during *in-vitro* seed colonization of *Aspergillus flavus* in groundnut. (**a**) Temporal distribution of relative abundance in expressed genes at different stages of fungal infection. The relative expression is found to be higher in infected samples of J 11 (J11-I) as compared to their control counter parts (J11-C) at 2^nd^ day after inoculation (2DAI). The expression level of infected samples of JL 24 (JL24-I) was higher as compared to JL24-C. (**b**) Relative abundance of all the genes expressed during *in-vitro* seed colonization across subgenomes of groundnut progenitors. The relative abundance in terms of FPKM values were mapped onto A10 and B07 of A and B subgenomes, respectively. (**c**) Number of differentially expressed genes-DEGs (induced and repressed) during *in-vitro* seed colonization. The highest number of DEGs were mapped onto A03 and B03 of A and B subgenomes, respectively.

From differential expression analysis, a total 4,445 DEGs were identified in different combinations across four stages (Supplementary Table 2). A total of 1,194 DEGs were identified when J 11 was compared with its control counterparts. Similarly, 930 DEGs were observed when infected JL 24 was compared with the control samples and 995 DEGs were identified when infected J 11 samples were compared with infected JL 24. The highest number of DEGs were observed during 1DAI (1,283), followed by 7DAI (1,141), 3DAI (1,061) and 2DAI (960). Upon comparing infected samples of J 11 with JL 24, the number of upregulated DEGs (640) were higher than down-regulated genes (355) in both the subgenomes. Among all the DEGs, most abundant genes expressed included senescence-associated proteins, resveratrol synthase, seed linoleate 9s-lipoxygenases (9s-LOX), Mlp like protein 43, pathogenesis-related (PR) proteins, peroxidases, glutathione-S-transferase, chalcone synthase, defensins, chitinases, etc. The relative abundance of expression of DEGs at 2DAI were higher as compared to other days (Fig. 2a).

The relative expression of all DEGs across different stages in control and infected samples of J 11 and JL 24 are presented in Supplementary Table 3. Senescence-associated proteins were found to be highly expressed at 1DAI in both J 11 and JL 24, though the level of expression was higher in JL 24. The expression of resveratrol synthase was found to be pathogen-induced as in control samples there was no significant level of expression of this gene. The level of expression was higher at 3DAI in J 11 as compared to its control counterpart. Interestingly, no significant difference was found when infected samples of J 11 and JL 24 were compared. The 9s-LOX were found be induced at 1DAI and flared up at 7DAI in J 11, whereas, its expression was repressed from 1DAI through 7DAI in JL 24. Similarly, Mlp-like protein 43 was repressed in infected samples as compared to control during initial days and its expression shot up in case of J 11 at 7DAI. PR proteins were found highly expressed after infection in case of J 11, though there was a considerable expression of these genes in JL 24 samples as well. Among the PR proteins, PR-10 was found to be in abundant during *Aspergillus* infection and expression was more at 3DAI. However, PR-like proteins were found to be accumulated in uninfected samples as well.

Peroxidases were another class of enzymes that were induced during infection in case of J 11 as compared to JL 24 and lignin-forming anionic peroxidases and cationic peroxidases were found in the initial period of infection i.e., 1DAI and 2DAI and other peroxidase classes like peroxidase 3, 10, 15, 16, 21, 44 etc. were found to be highly expressed at 7DAI in case of J 11. Glutathione-S-transferases (GST) were found to be induced in case of J 11 and clear repression was found in the case of JL 24 at 3DAI. Moderate expression was found during initiation of pathogenesis (1DAI and 2DAI) and limited expression was found during 7DAI in J 11. Similarly, chalcone synthases (CHS) and isomerases and isoflavone reductases were also found to be induced during infection in J 11 and repressed in JL 24 at 3DAI. Chitinase enzymes were found to be upregulated in J 11 at 7DAI and downregulated in JL 24 at 2DAI. Similarly, defensin enzymes and Phenylalanine ammonia lyase (PAL) related transmembrane protein-9 were found to be highly expressed in the case of J 11 at 3DAI as compared to their controls. The expression of heat shock proteins was also found to be downregulated in JL 24 at 3DAI. The ethylene-responsive transcription factor (ERF) proteins were upregulated at 1DAI and repressed at 2DAI and 3DAI in the case of JL 24. Microsomal omega-3 fatty acid desaturase was found to be upregulated in the case of infected samples of JL 24 at 3DAI as compared to J 11. Other genes like Kunitz-type trypsin inhibitor, desiccation protectant late embryogenesis abundant (LEA) 14 proteins, pectate lyase, subtilisin-like proteases, expansin, and serine-threonine protein kinases were found to be upregulated at 7DAI in J 11 as compared to JL 24. Also, the allergen genes (*Ara h 2*) were found to be repressed in J 11.

An important observation was made in case of genes involved in oxylipin production that are known to be involved in signal transduction i.e., LOX, peroxygenase 1 like proteins, 4-hydroxy-3-methylbut-2-en-1-yl diphosphate, phytyltransferases. All these genes were found to be marginally induced initially in JL 24 and repressed at the later stages. In J 11, the expression of LOX and peroxygenase was marginally decreased at 1DAI but increased at 7DAI. The expression of other two genes got induced at 3DAI in J 11.

### Genome-wide distribution of differentially expressed genes

A total of 19,398 and 20,227 genes were mapped to the pseudomolecules of A and B subgenomes, respectively. A total of 2,811 genes were mapped on A03, followed by 2,772 on B03. Among all, A07 (1,504) and B02 (1,660) had minimum number of genes mapped. The relative gene abundance, calculated from all the genes expressed in J 11 and JL 24 under control and infected conditions were plotted on A and B subgenomes. Interestingly, relative abundance of expression of genes was significantly higher in the case of pseudomolecule A10 (2.6 million reads) and B07 (2.3 million reads) of the A and B subgenomes, respectively. The frequency of mapped-reads was higher in infected samples compared to their control counterparts. The results clearly showed an increase in the level of expression in the case of J 11 during infection as compared to JL 24 (Fig. 2b). When the relative abundance of DEGs was checked, the DEGs were distributed across the groundnut genome and was higher in case of A10 pseudomolecule and distributed across A01, A10, B04 and B07 pseudomolecules (Supplementary Fig. 1).

Out of 4,445 DEGs identified, 2,007 DEGs (45.15%) were mapped on A subgenome and 2,438 (54.84%) DEGs on the B subgenome. These DEGs were distributed across pseudomolecules and the highest number of DEGs (376) were present on pseudomolecule B03 followed by A03 (313) and lowest number of DEGs were located on pseudomolecule A07 (143) with an average of 220 DEGs per pseudomolecule (Fig. 2c).

### Gene ontology (GO) and pathway analysis of differentially expressed genes

GO analysis annotated a total of 1,412 DEGs (70.35% of 2,007 DEGs in A subgenome) and 1,647 DEGs (67.55% of 2,438 DEGs in B subgenome) (Supplementary Fig. 2). Most of the DEGs were assigned to “Biological process” (3,321; 55%) followed by “Molecular function” (1,511; 25.33%) and “Cellular component” (1,132; 18.98%) on the A subgenome. Similarly functional categorization of the DEGs on B subgenome were assigned to “Biological process” (3,666; 55.27%) followed by, “Molecular function” (1,675; 25.25%) and “Cellular component” (1,291; 19.46%). Majority of the DEGs were related to response to stress/stimulus, signal transduction mechanisms, secondary metabolite mechanisms and lipid metabolism processes. These DEGs were more often related to molecular functions including binding, catalytic, hydrolytic and transporter activities and localized in cellular, membrane and intracellular part of the cells.

The pathway based analysis was done to further understand the molecular mechanisms of the differentially expressed genes. The DEGs were found to represent 119 and 171 pathways when mapped to A and B subgenomes, respectively and 89 non-redundant pathways were affected or influenced by their expression (Supplementary Table 4).

Majority of the pathways could be assigned to three general categories: (1) Primary metabolism including carbohydrate, lipid, amino acid and vitamin metabolisms. Carbohydrate metabolism pathways that are influenced in the present study include: citrate cycle, starch and sucrose metabolism, glycolysis, gluconeogenesis, galactose metabolism, etc. Similarly, lipid metabolism pathways like fatty acid biosynthesis and degradation, glycerolipid metabolism, sphingolipid metabolism, etc. were affected. Furthermore, amino acid metabolism pathways such as biosynthesis of phenylalanine, tryptophan, tyrosine, glutathione, etc. and several pathways related to the metabolism of vitamins were affected. (2) Secondary metabolic pathways dependent upon biosynthesis of terpenoids including biosynthesis of zeatin, ubiquinone, terpenoid-quinone, steroid, carotenoid, flavonoid biosynthesis, cutin, suberin, wax, etc. (3) Shikimate derivative dependent pathways including biosynthesis phenylpropanoid, stilbenoid, diarylheptanoid, gingerol, flavonoid, isoflavonoid, cyanoamino acid metabolism, etc.

### Differentially co-expressed gene modules

**Figure 3:**
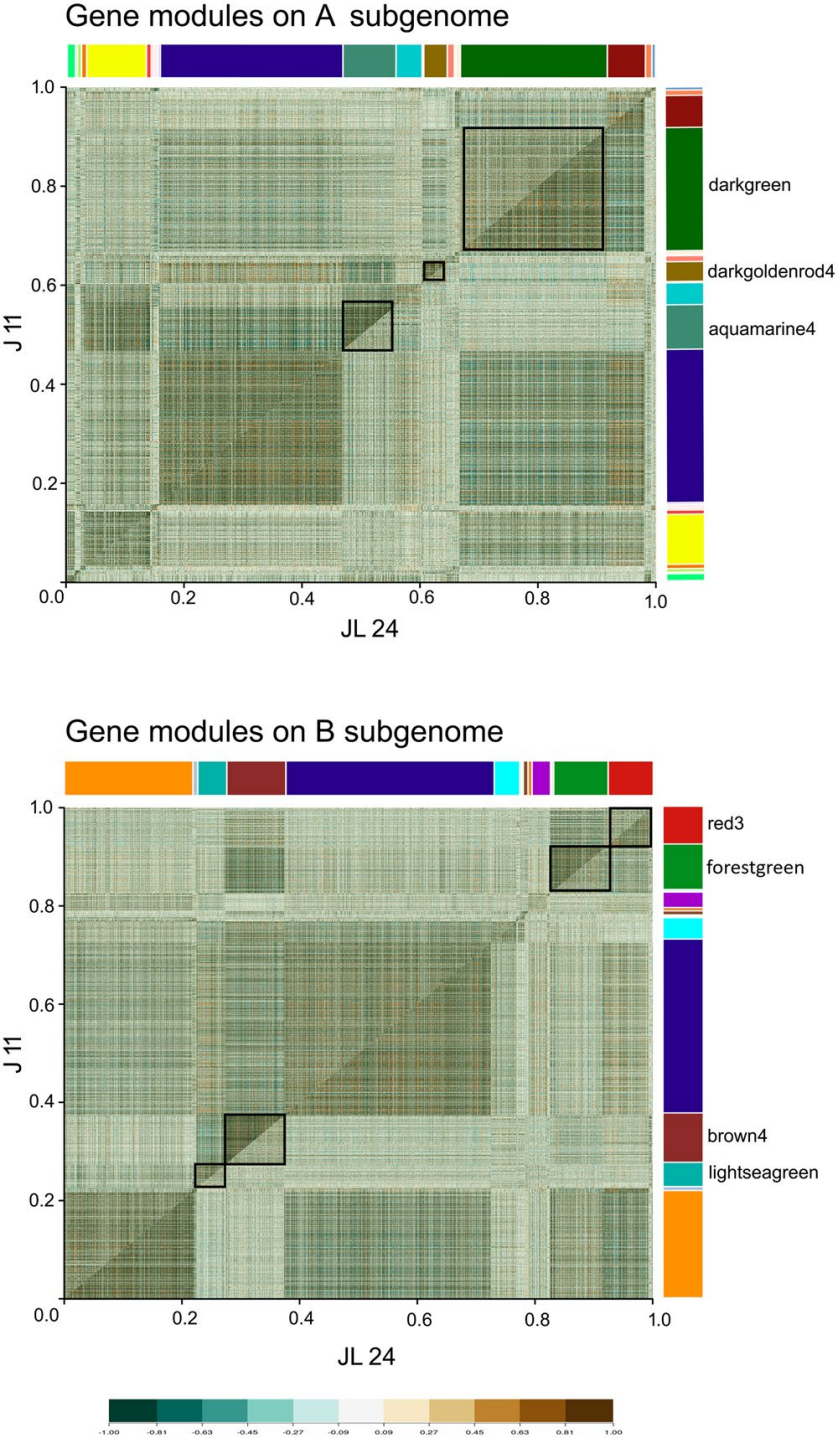
Differentially co-expressed modules between J 11 and JL 24 in A and B subgenomes of groundnut. The comparative correlation heat maps with brown color corresponding to positive correlation and blue corresponding to negative correlations are depicted in the figure. The upper diagonal of the main matrix shows a correlation between pairs of genes among J 11 (resistant) transcripts while the lower diagonal of the heat map shows a correlation between the same gene pairs in the JL 24 (susceptible). Modules are identified in the heat map by different built-in color bars on the right side of the heat map. Distinguished gene modules are demarcated by black squares.

DiffCoEx outlined differentially co-expressed modules between J 11 and JL 24 genotypes by grouping genes according to their shared, yet sophisticated, varied differential correlation patterns. Altogether, the analysis suggested significant differences between both the genotypes leading to the identification of 23 and 18 modules with different built-in color codes comprising of 19,251 and 19,923 genes in case of A and B subgenomes, respectively (Fig. 3). Three of these modules (darkgreen- 4707 genes; darkgoldenrod4-4755 genes; aquamarine4-1700 genes) were significantly highly correlated in the case of J 11 on A subgenome. In the case of B subgenome, three modules (forestgreen- 1808 genes; brown4-1960 genes; lightseagreeen-967 genes) were significantly more highly correlated in J 11 genotype and one module (red3-1500 genes) was found to be highly expressed and correlated in JL 24. Two interesting modules in each of the genomes *viz*., darkgoldenrod4 from A subgenome and red3 from B subgenomes were discussed here. The darkgoldenrod4 module from A subgenome comprised of the genes encoding plant defense such as PR proteins, chalcone synthase, chalcone-flavanone isomerase, resveratrol synthase, GST, senescence-associated proteins, WRKY, bHLH, leucine-rich repeat (LRR), chitinases, LEA proteins, calmodulin binding and transporting proteins and so on. The red3 module from B subgenome has genes encoding the binding and transporter activities like ABC (ATP binding cassette) transporters, plasma-membrane associated cation-binding protein, sugar transporters (SWEET), SNARE interacting proteins, autophagy related proteins, patatin like proteins and so on.

### Marker development and validation

**Figure 4:**
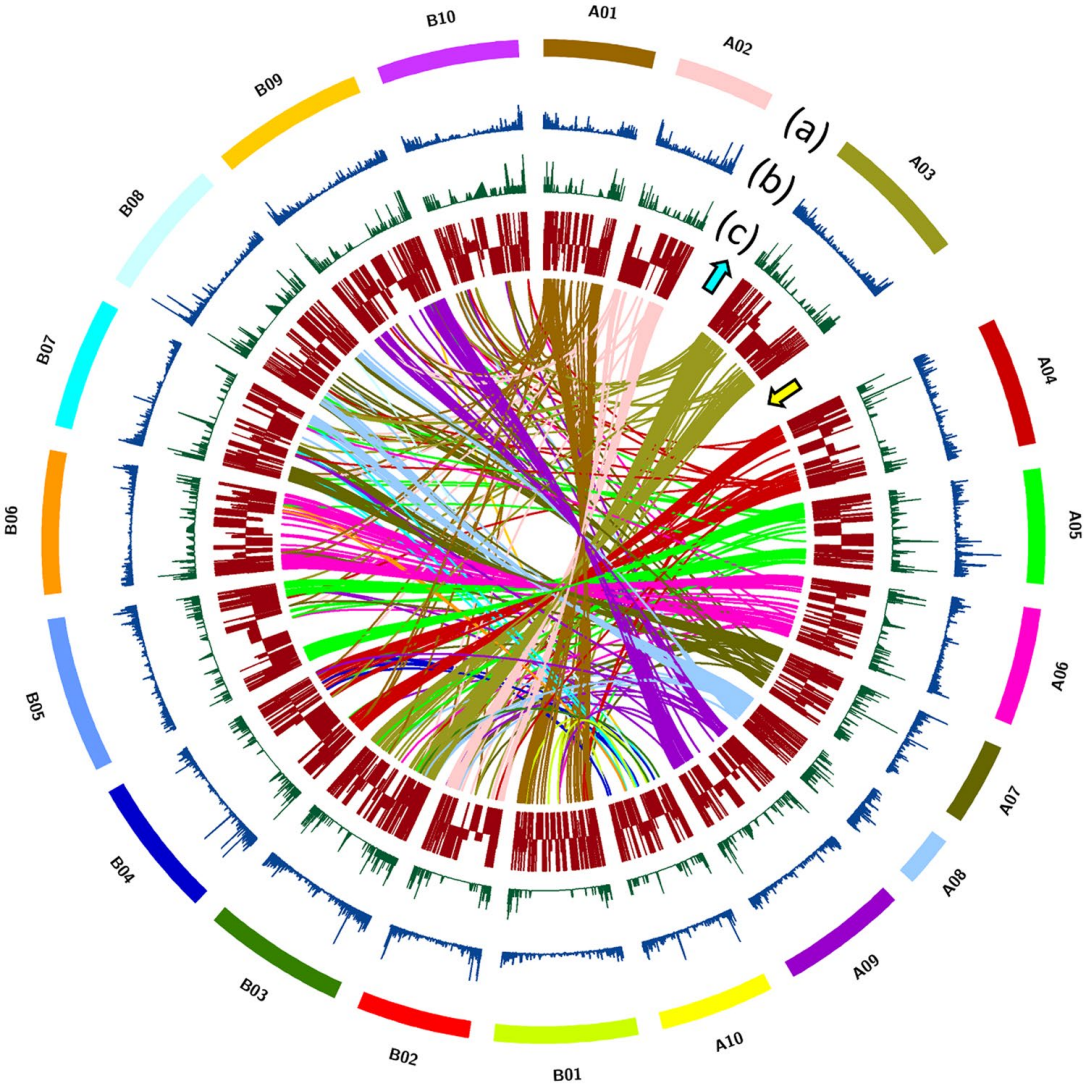
Distribution of single nucleotide polymorphisms (SNPs), Indels and differentially expressed genes (DEGs) across the pseudomolecules of progenitor reference genomes of groundnut. (**a**) Groundnut pseudomolecules from A subgenome are depicted as A01 to A10 and that of B subgenome are depicted as B01 to B10 (**b**) SNP density depicted in blue (**c**) InDel density depicted in green (**d**) both upregulated (with upward arrow) and downregulated (with downward arrow) differentially expressed genes depicted in brown. Synteny of the differentially expressed genes among the pseudomolecules are depicted with the different colored lines with respect to representative colors of the pseudomolecules.

A total of 39,742 and 40,946 sequence variants were identified between J 11 and JL 24 when aligned on A and B subgenomes, respectively. The variants on ‘A’ subgenome included 994 homozygous SNPs, 85 Indels and 38,663 heterozygous SNPs and Indels, whereas on ‘B’ genome included 1,651 homozygous SNPs, 72 Indels, and 39,223 heterozygous SNPs/Indels. Of the total SNPs, 50 and 118 SNPs/Indels were present in the 98 unique DEGs collectively from both A (30) and B (68) subgenomes, respectively. A maximum number of sequence variants were located on ‘B’ subgenome- B10 (22), followed by B02 (18), B05 (16) and B03 (15). The number of sequence variants was comparatively lesser in the case of ‘A’ subgenome, the highest being on pseudomolecule A05 (10). All 168 sequence variants were checked for their effect on the function of the gene (Supplementary Table 5). Maximum number of SNPs were non-synonymous coding (62) followed by synonymous coding (51), intronic (23), UTR_3_prime (12), UTR_5_prime (11), stop_gained (2), stop_lost (1) and others (5) when in case of both ‘A’ and ‘B’ subgenomes together (Table 2). About 5,025 selected SNPs from the available transcriptome were considered while designing an SNP array (58 K) chip in groundnut for further use in molecular breeding^34^. The identified sequence variants (SNPs and Indels) on DEGs across pseudomolecules, similarity relation of DEGs in both subgenomes have been shown in the Fig. 4. A total of 73 allele-specific primer pairs were designed to validate the sequence variants between J 11 and JL 24 (Supplementary Table 6). Nineteen polymorphic markers between J 11 and JL 24 were identified in genes like resveratrol synthase, calmodulin, GST, flavonoid glucosyltransferase, disease resistance proteins rpp13 and TIR-NBS-LRR (Supplementary Fig. 3). After thorough screening and validation, these functional/expression markers can further be used in genomics-enabled aflatoxin resistance breeding.

**Table 2:** Distribution of SNPs in differentially expressed genes across A and B subgenomes of groundnut.

SNP categories	A subgenome	B subgenome	Total
Non-synonymous coding	20	42	62
Synonymous coding	18	33	51
Intron	4	19	23
UTR_3 prime	2	10	12
UTR_5 prime	2	9	11
Others	3	2	5
Stop_gained	0	2	2
Frame shift	1	0	1
Stop_lost	0	1	1
Total	50	118	168

**Figure 5:**
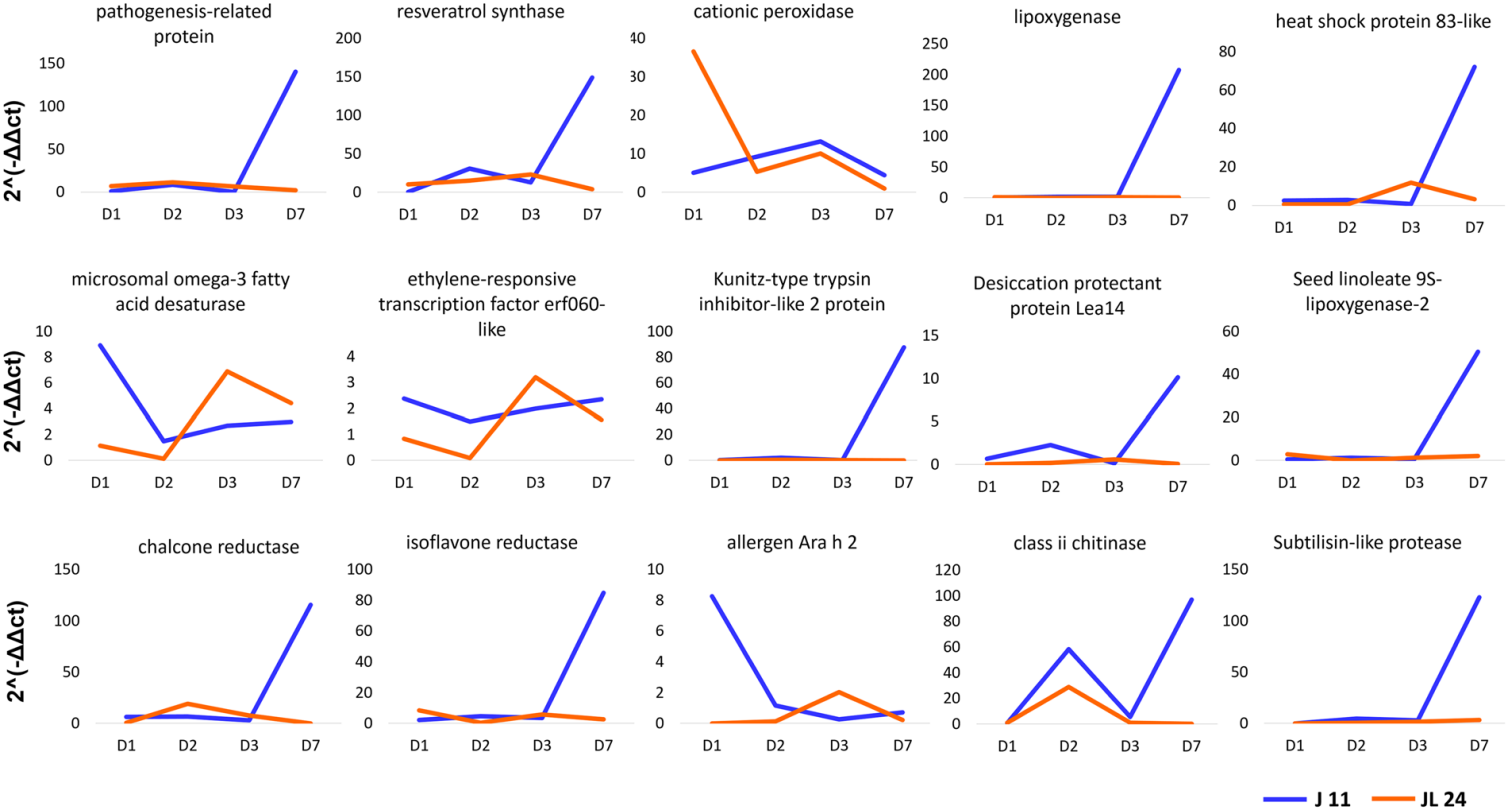
Validation of selected genes through qRT-PCR across different time periods of *Aspergillus* infection in J 11 (resistant) and JL 24 (susceptible) genotypes. Relative gene expression for 15 selected candidate genes like pathogenesis-related protein, resveratrol synthase, cationic peroxidase, lipoxygenase, heat shock protein 83-like, microsomal omega-3 fatty acid desaturase, ethylene-responsive transcription factor erf060-like, Kunitz-type trypsin inhibitor-like 2 protein, desiccation protectant protein Lea14, seed linoleate 9s-lipoxygenase-2, chalcone reductase, isoflavone reductase, allergen Ara h 2, class ii chitinase and subtilisin-like protease was calculated at four different time points of infection in case of J 11 and JL 24.

Validation of 15 DEGs including PR proteins, resveratrol synthase, cationic peroxidases, heat shock proteins-83 like, microsomal omega-3 fatty acid desaturase, ERF060, lipoxygenases, Kunitz type trypsin inhibitor, desiccation protectant LEA protein, seed linoleate 9s- lipoxygenases, chalcone reductase, isoflavone reductase, allergen Ara h2, chitinases and subtilisin like protease was carried out. qRT-PCR plots were drawn that indicated relative expression of these genes in case of J 11 and JL 24 (Fig. 5). The majority of the genes showed the similar pattern of expression as indicated by RNA seq results. There was slight variation in the expression of resveratrol synthase and ERF060 at the different time period of the infection though the overall expression was unvaried.

### Differentially expressed genes (DEGs) in fungus during infection

Expression analysis of unmapped groundnut transcripts on *Aspergillus* genome indicated differential expression of 578 genes consisting of 390 induced and 188 repressed genes. Interestingly, number of induced genes were more in infected JL 24 sample as compared to J 11 at 3DAI and that of repressed were more at 2DAI. The list included several genes like cytochrome p450 monooxygenases, conidial hydrophobin, cytochrome b5, O-methyl transferase (O-MTA), oxidoreductases, Ver 1, aflE norA aad adh-2 NOR reductase dehydrogenase, aflD nor 1 reductase and several transporter proteins.

The highest number of DEGs (113) were found to be induced in the case of 3DAI followed by 1DAI in case of JL 24 infected samples (compatible host-pathogen interaction). However, in the case of J 11 infected samples (incompatible interaction), highest number of DEGs (125) were induced at 2DAI (Supplementary Table 7). In compatible interaction, at the initiation of pathogenesis (1DAI), the genes involved in fatty acid biosynthesis (stearic acid desaturase, acyl-binding family, fatty acid elongase Gns1), cell-wall related (hydrophobin, chitin binding, pectate lyase), growth related (early growth response, conidiation-specific, conidial hydrophobin, developmental regulator), transporters [ABC transporters, major facilitator superfamily (MFS) transporters], and kinases (calcium calmodulin-dependent kinases, phosphofructokinases) were found to be induced. There was no or meager expression of these genes in incompatible interaction. At 2DAI, the majority of DEGs belonged to metabolic processes such as transcription with metal/ion binding functions were expressed. The genes that are involved in aflatoxin biosynthesis *viz*., Ver-1, aflE norA aad adh-2 NOR reductase dehydrogenase, aflD nor-1 reductase, Noranthrone monooxygenase and that of peroxidase activity (thioredoxin reductase, cytochrome p450, GST, glutathione peroxidase, glutaredoxin) were found to be highly upregulated in compatible interaction at 3DAI. Besides, the proteins that are involved in endocytosis and signaling *viz*., synaptobrevin (member of SNARE complex involved in vesicular trafficking and endocytosis), and dynamin-like proteins were induced at 3DAI in compatible interaction. Interestingly majority of these enzymes were integral part of the membranes and have metal/ion binding and transferase functions as per the gene ontology studies. During incompatible interaction, the genes involved in transport (phthalate transporter, uridine permease), hydrolysis (glucanases, ureidoglycolate hydrolase, epoxide hydrolase), proteolysis (serine peptidase, metalloendopeptidase, cupin domain) and oxidation-reduction reactions (xanthine dehydrogenase, lignostilbene dioxygenase, etc.) were upregulated as compared to the compatible reactions especially at 2DAI. The genes involved in aflatoxin biosynthesis were not observed in incompatible interaction.

## Discussion

Groundnut is a crop of global importance and aflatoxin contamination is the major bottleneck for international trade and industry, in addition to posing a health risk. Although sources of resistance to aflatoxin contamination in groundnut have been identified but there is no proper understanding of the molecular mechanisms associated with aflatoxin resistance. In this context, the present study provides a better understanding of the molecular mechanisms involved with resistance to IVSC through transcriptomic profiling of the crucial stages of fungal growth and infection. The mycelial growth and amount of toxin estimated on the 3^rd^ day (3DAI) were exceptionally high in JL 24 as compared to J 11. This period is considered to be the invasive growth for *Aspergillus* during infection and also in few other fungi like rice blast fungus^35^.

**Figure 6:**
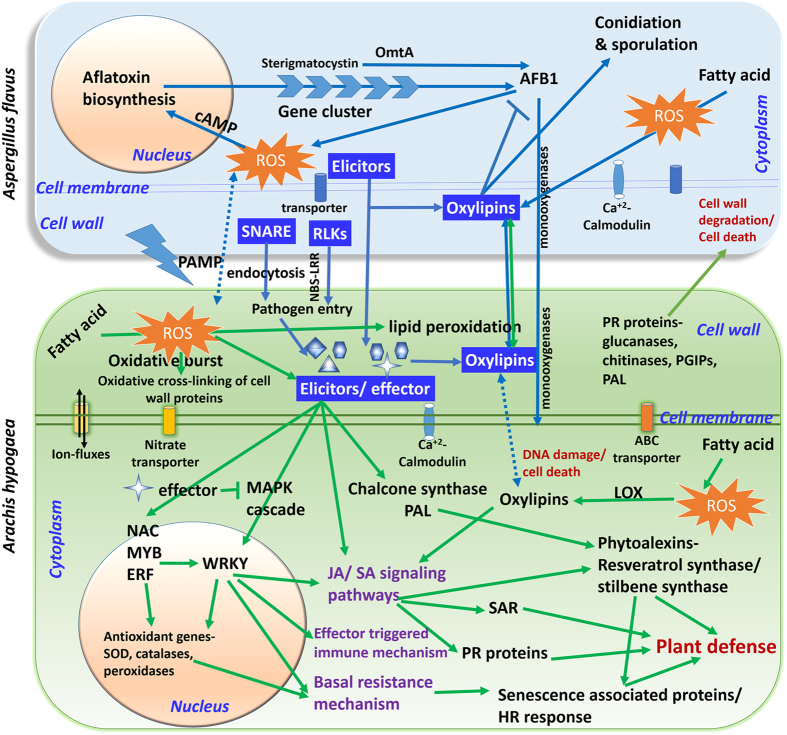
Illustration of cross-talk between groundnut (*Arachis hypogaea*) and fungus (*Aspergillus flavus*) during *in-vitro* seed colonization. The genes/components illustrated represent the defense responsive molecules identified from groundnut-*A. flavus* interaction during *in-vitro* seed colonization studies. The components marked within the green rectangular box (with green arrows) are those induced in groundnut and those which are in light blue rectangular box (blue arrows) are the components induced in pathogen in compatible reaction through mixed transcriptome analysis. The components represented in dark blue boxes have important role in host-pathogen interaction in this study. Briefly, the pathogenesis initiation occurs at plant cell wall where NBS-LRR, elicitors and oxylipins have important role in host-pathogen interactions. JA and SA signaling pathways mediated by transcription factors like WRKY, NAC, MYB and ERFs play important role in plant defense. During the process of defense the phytoalexins like resveratrol synthase/stillbene synthase, PR proteins, LOX, chalcone synthase and PAL were expressed. Basal resistance works with the expression of senescence associated proteins that give hypersensitive response to combat the pathogen entry further. The chitinases, glucanases, PGIPs, PAL, PR proteins induce cell wall degradation of fungi during pathogen entry. Due to pathogen attack, there is oxidative burst in the plant cell that regulates the oxidation of fatty acids that in turn involved in signal transduction. Transcriptome analysis of fungus indicated that the RLKs, SNARE complex, elicitors and oxylipins play important role in plant recognition and infection. Most of these proteins are integral part of cell wall and membrane. Genes involved in fungal growth, aflatoxin synthesis and transport were highly expressed in *Aspergillus*. Abbreviations: ROS- reactive oxygen species; SOD-superoxide dismutase; PR-pathogenesisrelated proteins; HR-hypersensitive response; JA-Jasmonic acid; SA- Salicylic acid; SAR-Systemic acquired resistance; LOX-lipoxygenase; PAL- Phenylalanine ammonia-lyase; Transcription factors-NAC (NAM, ATAF1/2 and CUC2 domain proteins), ERF (Ethylene responsive factors); MAPK- Mitogen-activated protein kinases; SNARE- soluble N-ethylmaleimide sensitive factor attachment receptor; RLK- receptor-like kinase; cAMP- Cyclic adenosine monophosphate, OmtA- O-methyl transferase; AFB1-aflatoxin B1.

The plant defense response to IVSC resistance seems to be a multifaceted endeavor that includes the recognition of a pathogen, the activation of a number of genes that lead to changes in the plant cell wall, changes in ion flux across the plasma membrane, formation of reactive oxygen species (ROS), production of phytoalexins, PR related proteins that induce resistance against pathogen attack (Fig. 6). The defense mechanism involves the expression of several TFs, systemic acquired resistance (SAR), mediated by SA, JA and ethylene signaling pathways^36–38^.

The present study identified about 89 non-redundant pathways that were influenced by the DEGs. The primary metabolic pathways that were affected mainly include carbohydrate, fatty acid, and amino acid metabolisms. Glucose is the preferred carbon source of *A. flavus* hence, up-regulation of sucrose hydrolyzing enzymes that promote disease development by providing a steady supply of nutrients to the pathogen. Earlier studies also indicated that pathogens can manipulate plant’s metabolism to create a congenial environment for their growth^39, 40^. Starch degradation and hexose mobilization were reported in *A. flavus* infected maize^41^.

Oxygenase enzymes act on fatty acids and produce oxylipins that play important role in signaling pathways and conidial growth in fungi and also they exhibit a common structural motif in plant and pathogen^42, 43^. The role of fatty acids was also evident in aflatoxin production, as the first acting enzymes in the aflatoxin biosynthesis pathway are fatty acid synthases (*fsa1*, *fsa2*)^44^. These fatty acids also act as precursor molecules for the production of cuticular components and phytohormone JA that are involved in plant defense. The direct roles for fatty acids in plant defense by modulating the basal, effector-triggered and systemic immunity responses have also been demonstrated^45^. Secondary metabolic pathways that include terpenoids, flavonoids, and steroid biosynthesis pathways have also shown to be affected during the *Aspergillus* infection that play an important role in plant defense by anti-oxidative properies.

The flavonoids are known to quench ROS generated both by the pathogens and the plant as a result of the infection^46^. Biosynthetic pathway of flavonoids includes the synthesis of chalcone catalyzed by CHS and chalcone isomerase. In the present study, we observed an increased expression of CHS and chalcone-flavanone isomerase proteins under stress condition in J 11 as compared to JL 24. The role of CHS in SA driven signaling during plant defense is reported^47^. Phytoalexins especially resveratrol synthase/ stilbene synthase were the most abundant enzymes that were produced in response to *Aspergillus* infection in case of J 11 and JL 24 as compared to their control counterparts during early stages of infection. The level of expression was higher in the case of J 11 as compared to JL 24. Interestingly the control samples showed meager expression indicated the expression of these genes upon the trigger from pathogen attack. Accumulation of phytoalexins at post-pathogen or elicitor induction is shown in many plants including *Brassica* ^48^, chickpea^49^ and soybean^50^. In the case of groundnut, resveratrol, arachidin and arahypin phytoalexins were found to be induced when infected by *Aspergillus caelatus* ^51^. A recent report about the effect of resveratrol on *A. flavus* indicated decreased growth and aflatoxin production in fungus^52^. SA is derived from phenylpropanoid pathway, and their glucoside conjugates play important role in infection initiation. It was interesting to note in the current study that the salicylate o-methyltransferase, enzyme involved in SA pathway was found to be induced during 3DAI in J 11 and JL 24 cultivars in infected samples and level of expression was more in JL 24 as compared to J 11, indicating the hypersensitive responses. The enzymes like PAL and GST were found to be highly up-regulated in J 11. GSTs under stress have peroxidase activity that protects the cells from oxidative injuries^53^. Senescence-associated proteins, geraniol 8-hydroxylase, serine-threonine protein phosphatase, seed linoleate 9s-lipoxygenase (LOX) and PR proteins were the other important enzymes that were expressed at higher levels in J 11 compared to JL 24 during infection. The senescence-associated proteins were found to be expressed in J 11 and JL 24 at different time points, i.e., initial induction was seen in J 11 and later induction was seen in JL 24. This indicated the dual function of senescence-associated proteins that initially expressed in J 11 as a defense response to trigger basal immunity of plant by SAR and then the increased expression in JL 24 at later stages as seeds got invaded by fungus and initiates senescence. LOX enzymes catalyze the oxygenation of fatty acids to produce oxylipins and increased LOX activity may contribute to resistance by generating signal molecules such as JA, methyl-JA or lipid peroxidases^54^ that was illustrated in maize during *A. flavus* infection^55, 56^. There are controversial studies regarding the activity of LOX genes in resistance mechanism pertaining to *Aspergillus* infection in soybean^57^. Hence there is a need to study the effect of the LOX genes thoroughly in groundnut. The geraniol 8-hydroxylase involved in production of iridoid glycosides reduce the fungal growth was reported^58^. PR proteins are produced in plants upon pathogen attack and induce SAR that play important role in cell rescue and defense. These proteins include endoglucanases, chitinases, peroxidases, defensin proteins, heat shock proteins that either act on fungal cell wall or act as signaling molecules to trigger the basal resistance and pathogen associated molecular pattern (PAMP). These molecules also act on cross-linking of molecules of cell wall to deposit lignin, callose molecules that prevent the pathogen entry inside the plant/ seed^59^. In the present study, some PR-like proteins were also found to be accumulated in the both infected and uninfected samples. PR-like proteins were found to have role in development of plant tissues in a developmentally controlled manner besides their role in defense response to stresses^60^. LEA proteins were also over-expressed in infected samples, of *Arabidopsis* ^61^ and maize^62^, but limited information is available about their role in plant defense. The relation between aflatoxin and allergens is not very well established, but some reports showed abundance of allergens storage proteins during *A. flavus* infection in groundnut^63, 64^. However the relation between aflatoxins and allergens in groundnut need to be thoroughly understood.

The differential co-expression studies also indicated a correlation pattern between expression of genes in J 11 and JL 24 genotypes. In fact, when two differentially correlated gene modules that were highly expressed and correlated in resistant (darkgoldenrod4) and susceptible (red3) genotypes were studied, they tend to contain a bunch of genes that have interesting roles in plant-pathogen interactions. The gene modules that were highly expressed in resistant genotype contained defense related genes like disease resistance response proteins/ PR proteins, chalcone synthase, resveratrol synthase, GST, senescence- associated proteins etc. The genes that were highly expressed and correlated in susceptible genotype present in red3 modules were involved in pathogen recognition, binding, transporter activity. The high expression of SNARE interacting proteins in JL 24 and also in fungus suggests the presence of common motif/complex that is evolutionarily selected to ensure the survival of pathogens within the eukaryotic environment^65^. Genetic markers for screening aflatoxin resistance in groundnut are very limited and this study provided the much needed genetic markers (SNPs and Indels) which upon validation can be used in GAB for enhancing resistance to aflatoxin contamination.

The simultaneous analysis of mixed transcriptomes of groundnut- *Aspergillus* provided the insights into the host-pathogen interactions. Similar approach was successfully used to study rice-blast fungus^66^ and sorghum-leaf spot fungus^67^ interactions. In present study, the pathogen transcriptome analysis indicated expression of genes related to fungal growth, aflatoxin production and transport. The genes related to initiation of pathogenesis, fatty acid biosynthesis, conidiation, transportation were regulated at initial period (1DAI) and that of aflatoxin production, peroxidation process were upregulated at 3DAI in compatible interaction. This observation can be correlated to the higher level of aflatoxin estimated at 3DAI. The presence of transporters implies an important role in fungal nutrient uptake as well as in signal transduction. Most of transporter proteins detected in the present study were integral part of the membrane proteins that might play very important role in inter-kingdom cross-talks.

The initiation of pathogenesis starts with the recognition of receptor like kinases (RLK) by the plant cell for pathogen detection. In the present study, the expression of large number of RLKs *viz*., serine-threonine protein kinase (br1-like, nak types), wall-associated receptor kinase-like 9, LRR family protein, etc. were observed in both compatible and incompatible interactions. NBS-LRR genes are the TF binding genes involved in plant defense^68^. Mitogen activated protein kinase (MAPK) cascade activated by RLKs participate in innate or induced plant defense response by their interaction with WRKY TFs^69^. During pathogenesis there is out-burst of ROS that is utilized in lipid peroxidation. Hyper-oxidant status of cell is pre-requisite for the onset of aflatoxin biosynthesis and there seems a close link between peroxisome metabolism and aflatoxin synthesis in *A. flavus* ^70^.

In summary, the comparative analysis of transcriptome of resistant and susceptible cultivars during *Aspergillus* infection has provided insights into various genes involved in several pathways that coherently induce plant defense mechanisms through basal and induced resistance. Further the key candidate genes *viz*., resveratrol synthase, chalcone synthase, chitinases, 9s-LOX, PR proteins, GSTs can be used in groundnut improvement by either genomics-enabled breeding or modern transgenic approaches. Most importantly, in addition to greater insights on the genetic mechanism and discovery of candidate genes, this study also provided genetic markers for IVSC resistance in groundnut.

## Methods

### Materials and methods

#### Plant and fungal materials

The resistant (J 11) and susceptible (JL 24) genotypes of groundnut and highly toxigenic *A. flavus* strain AF 11-4, characterized at Groundnut Pathology Unit of ICRISAT, were used for conducting transcriptomic study for IVSC resistance. The strain in pure culture form was grown on Potato Dextrose Agar for 7 days and conidial suspension was prepared (1 × 10^6^ spores/ml).

#### Screening of *in-vitro* seed colonization

A total of ~200 healthy seeds for J 11 and JL 24 were surface sterilized with 0.1% HgCl_2_ for 3 min followed by three washes with sterile distilled water. Two different sets (control and infected) were made for each genotype. One set of sterilized seeds i.e., ~100 seeds for each genotypes were transferred to sterile filter papers on petri dishes and were used as control. The second set with remaining ~100 sterilized seeds were infected with the spore suspension of *A. flavus* toxigenic strain ‘AF 11-4’ with optimum concentration of 10^6^ colony forming units/ml. Both the sets were incubated in a moist chamber with 100% relative humidity at 28 °C in dark. The samples were taken at 1 day after inoculation (1DAI), 2DAI, 3DAI and 7DAI from the control and infected samples of J 11 and JL 24 genotypes. About 10-12 seeds at each time intervals were frozen in liquid N_2_ until further use and the remaining seeds were used for aflatoxin estimation and microscopic observation of seed coat. This experiment was carried out twice and each set was considered as independent biological replicates. In total, 16 samples (2 genotypes × 4 stages × 2 treatments) were analyzed for aflatoxin estimation and RNA isolation.

#### Aflatoxin quantification and microscopic observation of seed coat

Indirect competitive enzyme-linked immunosorbent assay (ELISA) using polyclonal antibodies produced against Aflatoxin B1 (AFB_1_) was used for quantitative estimation of total aflatoxins accumulated under control and infected treatments following the protocol explained by Waliyar *et al*.^71^. The seed coats of the control and infected genotypes stained with coomassie brilliant blue and were observed through Zeiss Axio Scope.A1 Florescence microscope (Carl Zeiss, Germany).

#### RNA isolation and sequencing

Total RNA was isolated from the seeds using “NucleoSpin^®^ RNA Plant” kit (Macherey-Nagel, Germany) following manufacturer’s protocol. RNA quality and quantity was determined using Nanodrop 1000 spectrophotometer (Thermo Fisher Scientific Inc, USA) and Bioanalyzer RNA Nano chip (Agilent Technologies, USA). Approximately 5 µg of total RNA pooled in equal quantity from two biological replicates were used for the construction of cDNA library using mRNA-Seq Sample Prep kit (Illumina Inc., USA) following manufacturer’s instructions. The RNA samples with 260/280 ratio of 1.8 to 2.1, 260/230 ratio of 2.0 to 2.3 and RIN (RNA integrity number) more than 7.0, were used for sequencing on Illumina HiSeq 2500 platform. The samples were sequenced as 2 × 100 bp paired-end reads. Filtered reads were obtained after running the quality control (QC) using NGS-QC box^72^.

#### Read alignment and gene expression estimation

The reads were mapped to pseudomolecule level genome assemblies^30^ using TopHat2^73^ using a reference GTF file using the −G option and the inner distance between the mating pairs was set to 180 bp with a mate standard deviation of 75. The reads that were not aligned to the reference genomes were used to align on the *Aspergillus* genome (*A. flavus* NRRL3357 GCF_000006275.2) available at NCBI^74^.

Read counts were normalized by calculating the fragments per kilobase of exon per million fragments mapped (FPKM) value for each genes. Reads were assembled into transfrags using Cufflinks v2.1.1^75^. Genes with FPKM ≥ 1 were further considered for analysis. Differentially expressed genes were identified using Cuffdiff^76^. The log_2_ fold change values of ≥ + 2 and ≤ − 2 (up- and down-regulated) with significance level ‘yes’ were considered for DEGs.

Functional categorization of the DEGs was studied using GoSlim (http://www.agbase.msstate.edu/cgi-bin/tools/goslimviewer_select.pl) while gene ontology (GO) terms were assigned to the expressed genes and assignment of KEGG pathways was carried out using Blast2GO^77^. DiffCoEx analysis method was used to identify differentially coexpressed gene modules based on Weighted Gene Coexpression Network Analysis (WGCNA) framework^78^. The clustering was done using standard hierarchical clustering with average linkage, followed by coexpressed gene modules detection from the resulting dendrogram, using a fixed cut height of 0.3, minimum module size of 30, and the soft thresholding power of 10. Each module was depicted with different built-in colors.

The alignment (bam) files were used for variant discovery with Genome Analysis Tool Kit (GATK)^79^. A position was reported as a variant for a genotype if phred quality score > 30 supported by a minimum read depth of 5. Distribution of DNA polymorphisms was assessed by calculating their frequency in a window size of 100 Kb along each pseudomolecule. For identification of effects of synonymous and non-synonymous SNPs and Indels, SnpEff program^80^ was used. In-house Perl scripts were used to analyze the distribution of the variations (SNPs and Indels) across the genome. BatchPrimer3 tool was used to design allele-specific primers.

#### Quantitative real time PCR (qRT-PCR) analysis

To validate the expression analysis of key candidate genes, primers were designed using Primer 3 plus tool (http://www.bioinformatics.nl/cgi-bin/primer3plus/primer3plus.cgi). cDNA was prepared using superscript first strand synthesis followed by second strand synthesis according to the instructions of manufactures (Invitrogen, USA). The primer pairs used for qRT-PCR are presented in Supplementary Table 8. The primer efficiency was checked using 10-fold dilution of cDNA and primers with efficiency ranging from 90–110% were used further for qRT-PCR. The qRT-PCR was performed on the Applied Biosystems 7500 Real-Time PCR systems using SYBRGreen chemistry following the manufacturer’s instructions (Invitrogen, USA). At least two independent biological replicates and three technical replicates at four different stages (1DAI, 2DAI, 3DAI and 7DAI) were used for qRT-PCR analysis. Alcohol dehydrogenase (*adh3*) was used as a reference gene and relative expression of the candidate genes was calculated using the delta delta Ct method^81^.

## Electronic supplementary material

Supplementary Information

Supplementary Table S3

Supplementary Table S4

Supplementary Table S5

Supplementary Table S6
