# Supplementary material for: Aspergillus flavus infection triggered immune responses and host-pathogen cross-talks in groundnut during in-vitro seed colonization
Source: Sci Rep. 2017 Aug 29;7:9659. doi: 10.1038/s41598-017-09260-8 (PMC5574979; doi:10.1038/s41598-017-09260-8)
Supplement: Supplementary file 1 — Supplementary Information [file 41598_2017_9260_MOESM1_ESM.doc]

## *Scientific Reports* supplementary Information

Article title: ***Aspergillus flavus* infection triggered immune responses and host-pathogen cross-talks in groundnut during *in-vitro* seed colonization**

Authors: Spurthi N Nayak, Gaurav Agarwal, Manish K Pandey, Hari K Sudini, Ashwin S Jayale, Shilp Purohit, Aarthi Desai, Liyun Wan, Baozhu Guo, Boshou Liao, Rajeev K Varshney

The following supplementary information is available for this article:
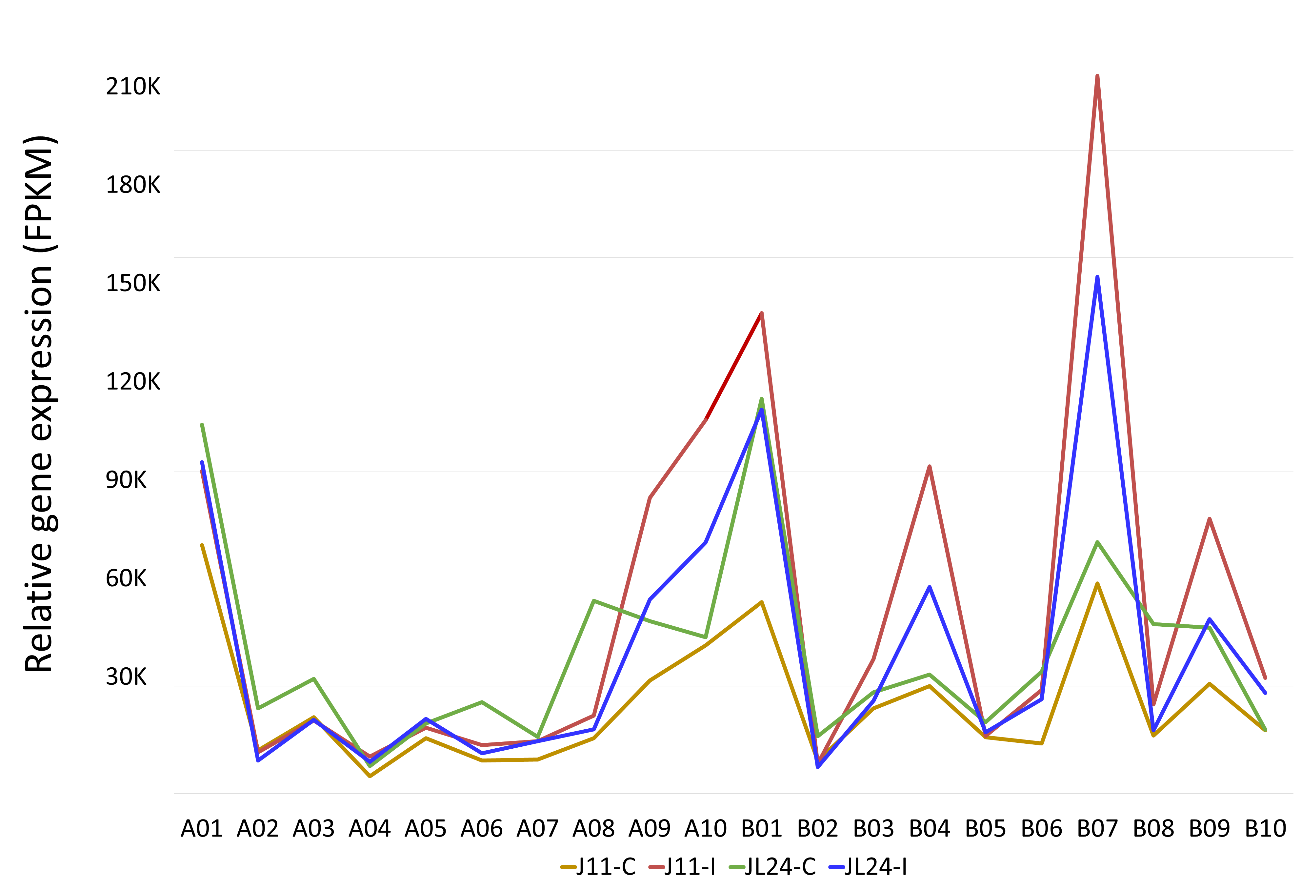


**Supplementary Figure S1.** Relative gene expression of differentially expressed genes across the twenty pseudomolecules of A and B subgenomes; FPKM-Fragments per kilobase of exon per million fragments mapped.


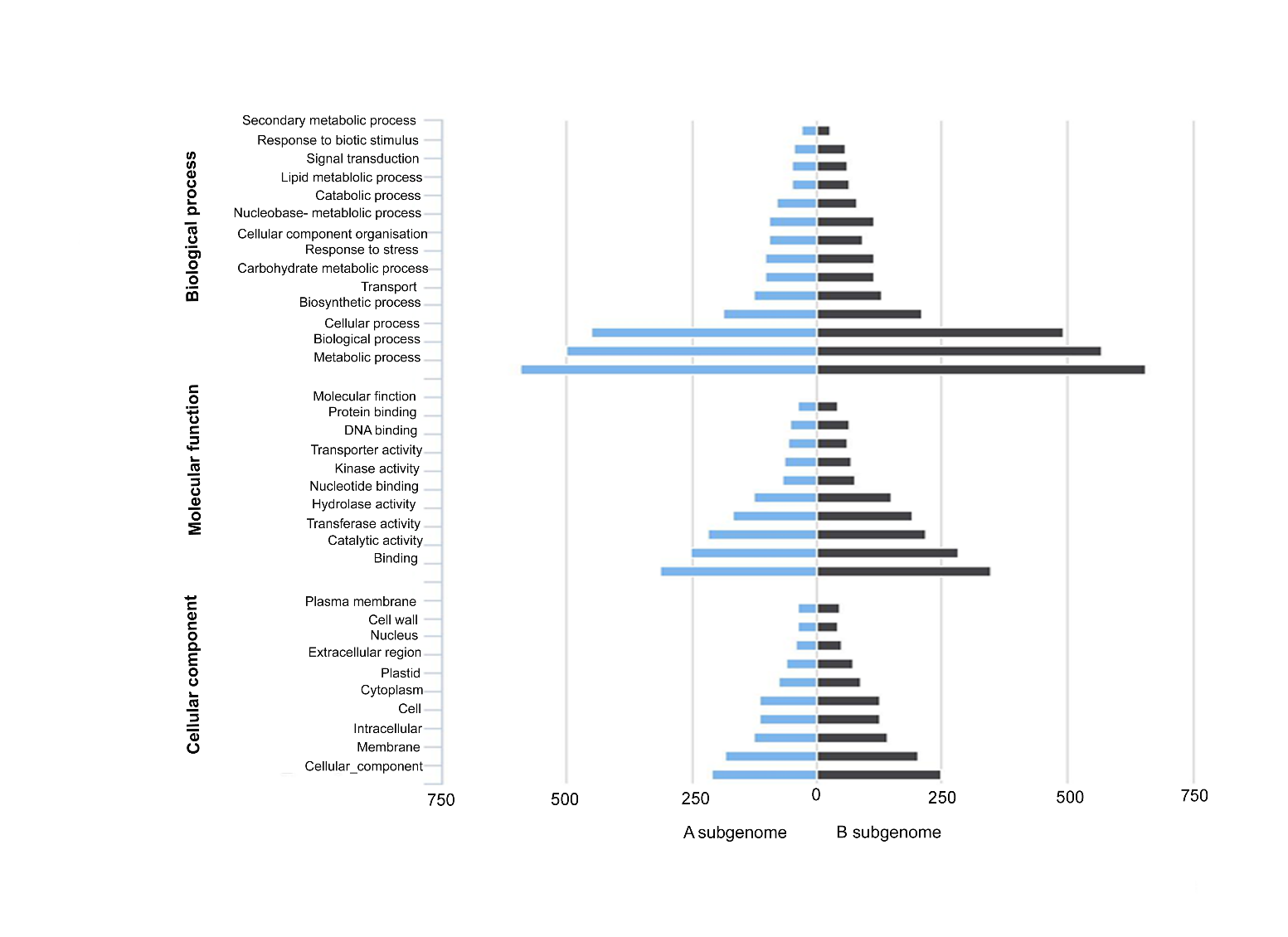


**Supplementary Figure S2.** Functional categorization of *Aspergillus* infection responsive genes during *in-vitro* seed colonization in groundnut into different gene ontology (GO) categories- Biological Process, Molecular Function, Cellular Component. The GO categories are represented in blue for A subgenome and black for B subgenome. The number of genes mapped to respective GOs are depicted in X-axis.


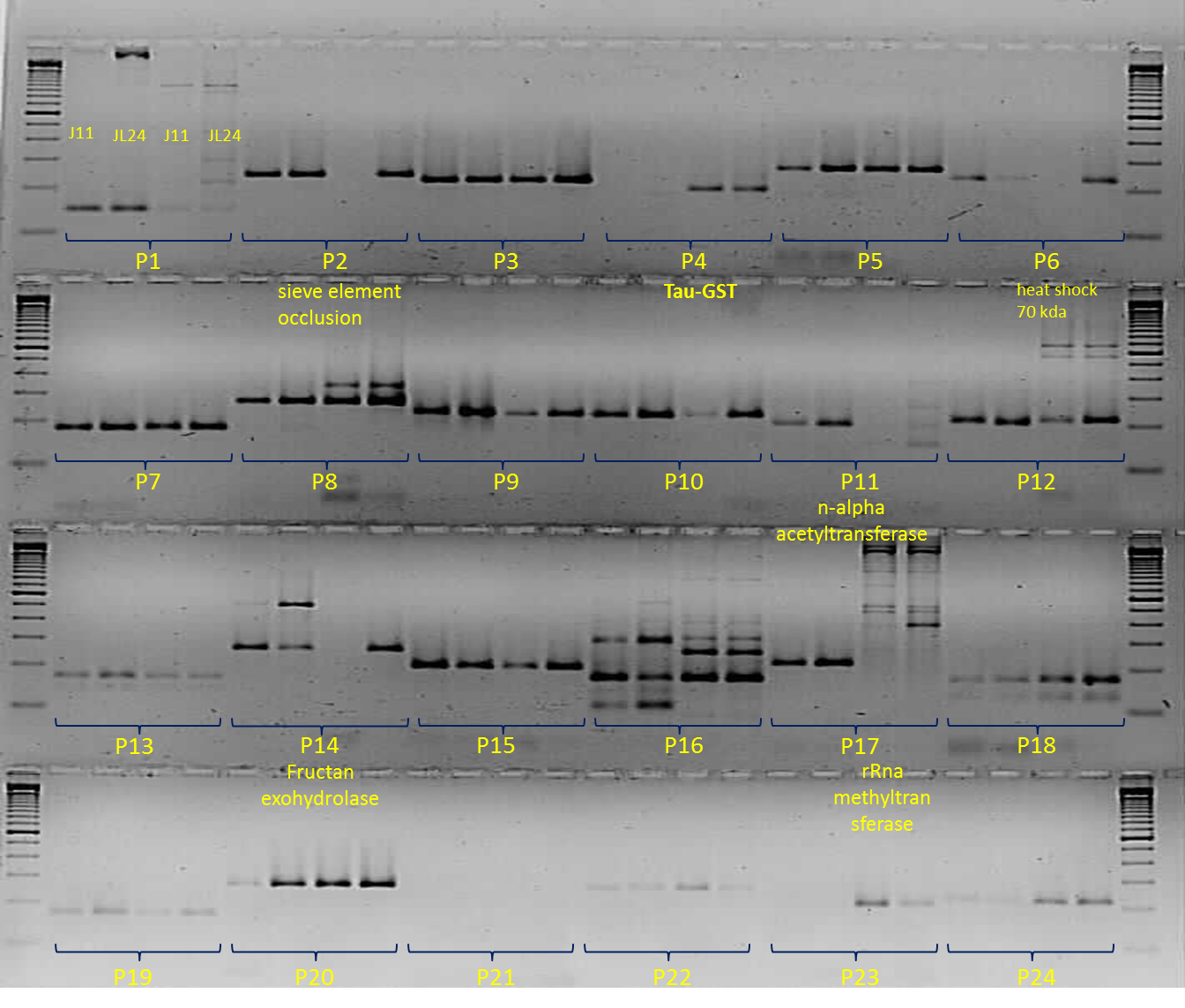


**Supplementary Figure S3 (a)**

**
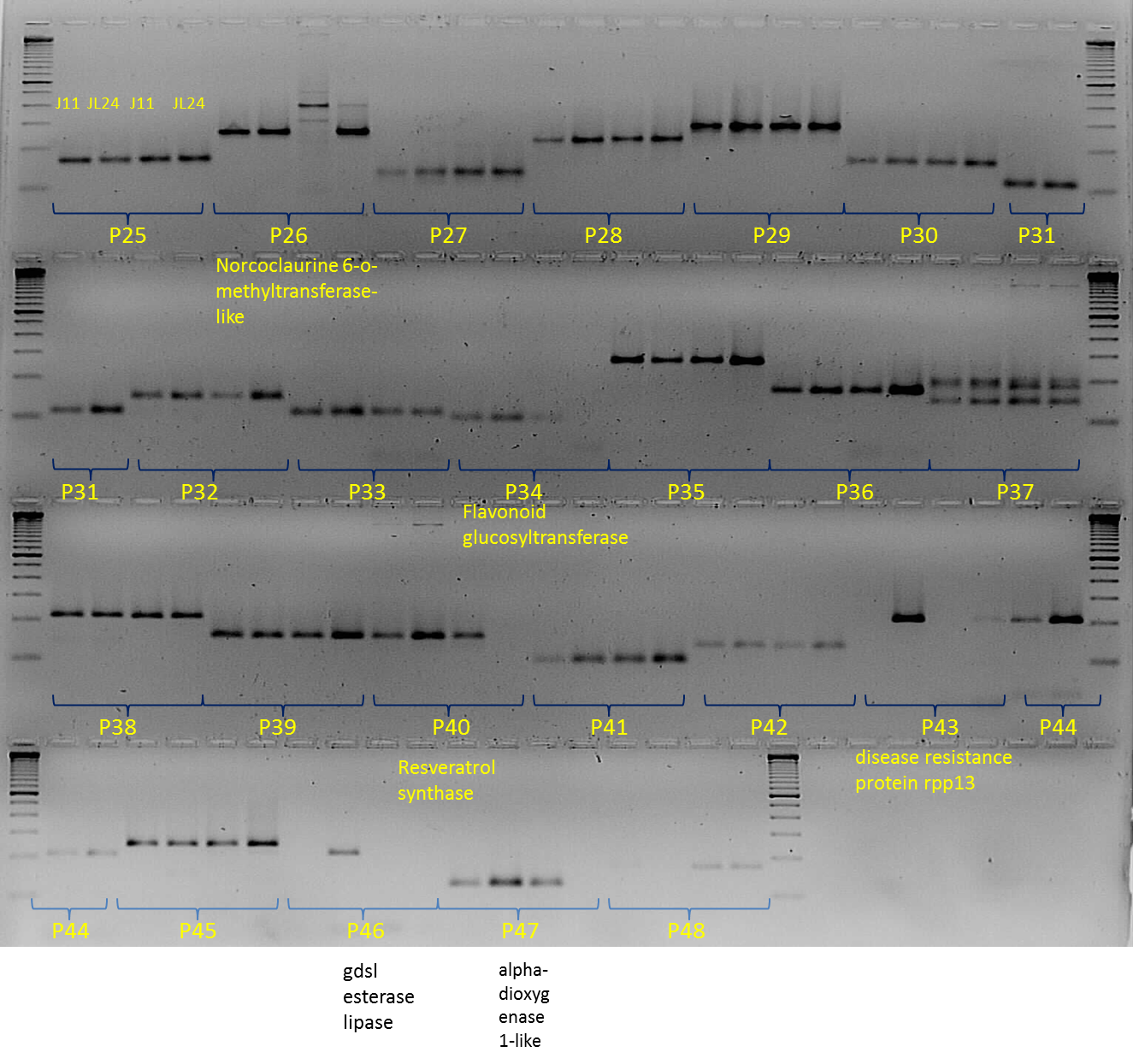
**

**Supplementary Figure S3 (b)**

**
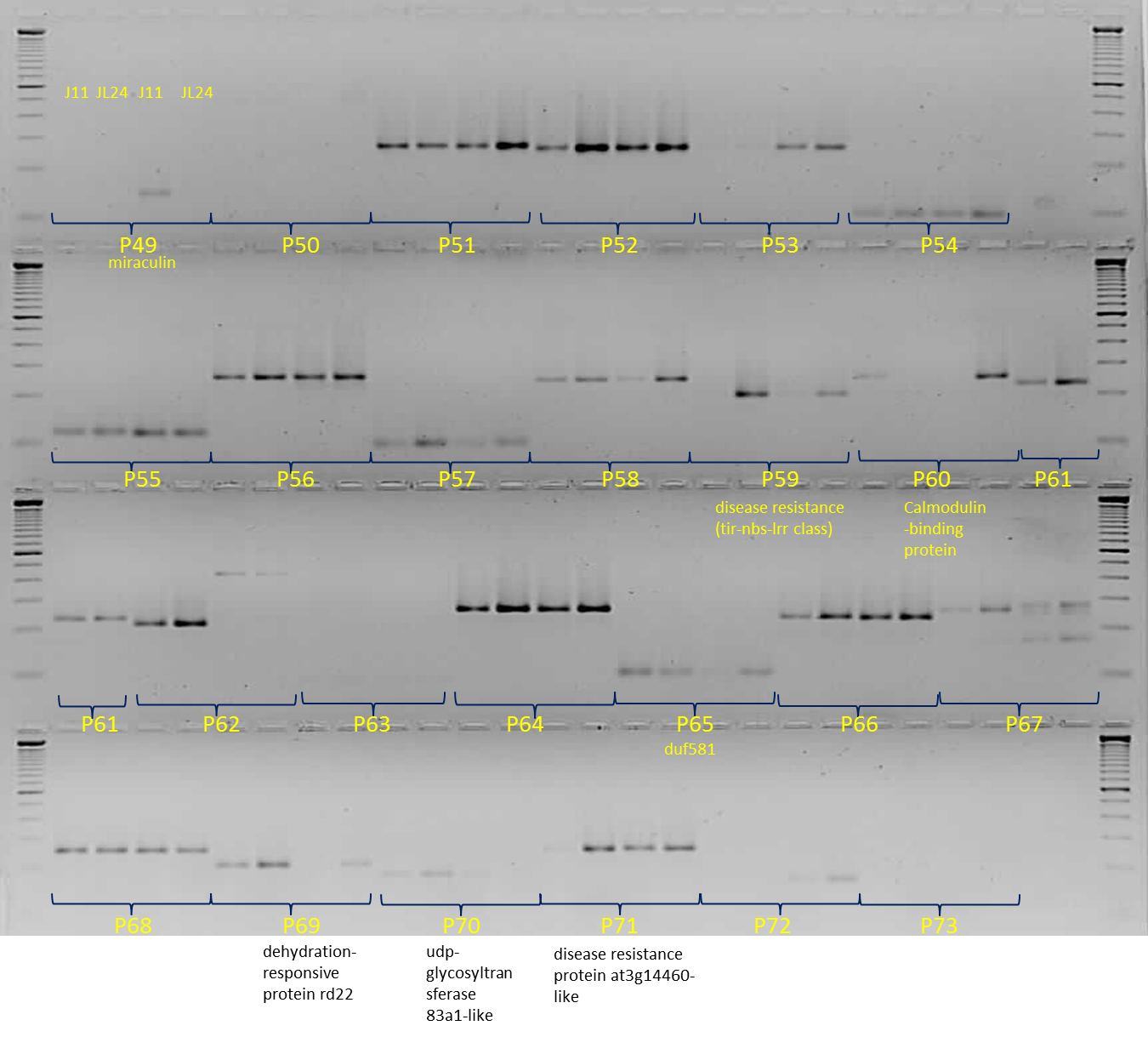
**

**Supplementary Figure S3 (c)**

**Supplementary Figure S3 (abc).**

Checking polymorphism of allele specific primers in J 11 and JL 24

Primers were designed for 73 DEGs of which 19 primer pairs gave polymorphism between J 11 and JL 24. The polymorphic markers are labelled with their annotation.

**Supplementary TableS1** Reads mapped on different genomic regions of *Arachis* proginators (A and B subgenomes)

| Total number of reads mapped on 'A' subgenome (million) | Reads mapped on exonic regions of 'A' subgenome | Reads mapped on intronic regions 'A' subgenome | Reads mapped on intergenic regions 'A' subgenome | Total number of reads mapped on 'B' subgenome (million) | Reads mapped on exonic regions of 'B' subgenome | Reads mapped on intronic regions 'B' subgenome | Reads mapped on intergenic regions 'B' subgenome |
| --- | --- | --- | --- | --- | --- | --- | --- |
| 64.26 | 52.56 | 3.85 | 6.30 | 64.93 | 52.92 | 3.89 | 6.46 |
| 54.39 | 41.14 | 3.78 | 7.42 | 55.16 | 41.92 | 3.90 | 6.99 |
| 53.49 | 40.96 | 3.57 | 6.87 | 54.31 | 41.78 | 3.67 | 6.51 |
| 47.57 | 32.72 | 3.58 | 4.91 | 51.30 | 38.09 | 3.44 | 5.31 |
| 44.70 | 35.29 | 2.77 | 4.32 | 46.05 | 36.76 | 2.82 | 4.64 |
| 33.87 | 19.58 | 2.44 | 3.42 | 37.78 | 26.49 | 2.39 | 3.65 |
| 43.69 | 31.30 | 3.15 | 5.52 | 45.45 | 33.86 | 3.20 | 5.35 |
| 41.59 | 28.24 | 3.29 | 5.21 | 44.02 | 31.79 | 3.28 | 5.28 |
| 54.70 | 42.05 | 3.54 | 5.10 | 57.00 | 45.05 | 3.53 | 5.48 |
| 57.22 | 45.57 | 3.65 | 5.95 | 58.23 | 46.52 | 3.70 | 6.08 |
| 52.46 | 39.49 | 3.73 | 6.65 | 53.69 | 40.76 | 3.84 | 6.68 |
| 47.70 | 34.06 | 3.55 | 4.61 | 51.13 | 38.33 | 3.44 | 5.45 |
| 48.19 | 34.53 | 3.43 | 4.66 | 51.40 | 38.90 | 3.41 | 5.14 |
| 52.27 | 36.11 | 3.53 | 5.27 | 55.76 | 41.94 | 3.34 | 5.58 |
| 44.34 | 35.65 | 2.80 | 4.73 | 44.64 | 35.72 | 2.79 | 4.75 |
| 43.71 | 31.28 | 3.34 | 4.80 | 45.72 | 34.27 | 3.39 | 5.14 |
| 784.17 | 580.55 | 54.02 | 85.73 | 816.55 | 625.13 | 54.03 | 88.49 |

Number of reads (in million) mapped on exonic, intronic and inter-genic regions when the filtered reads were mapped on A and B subgenomes are shown.

**Supplementary Table S2** Temporal distribution of gene expression at different stages of infection during *in-vitro* seed colonization in groundnut

| Subgenomes | induced/upregulated | 1DAI* | 2DAI* | 3DAI* | 7DAI* | Total |
| --- | --- | --- | --- | --- | --- | --- |
| A | J 11_control Vs J 11_infected | 41 | 18 | 113 | 193 | 365 |
| B | J 11_control Vs J 11_infected | 42 | 17 | 120 | 237 | 416 |
| A | JL 24_control Vs JL 24_infected | 57 | 2 | 20 | 45 | 124 |
| B | JL 24_control Vs JL 24_infected | 58 | 5 | 18 | 41 | 122 |
| A | JL 24_infected Vs J 11_infected | 65 | 43 | 41 | 133 | 282 |
| B | JL 24_infected Vs J 11_infected | 86 | 52 | 77 | 143 | 358 |
| A | J 11_control Vs JL 24_control | 152 | 76 | 141 | 20 | 389 |
| B | J 11_control Vs JL 24_control | 42 | 54 | 64 | 22 | 182 |
|  | Total (daywise) | 543 | 267 | 594 | 834 | 2238 |
|  |  |  |  |  |  |  |
| Subgenomes | Repressed/down regulated | 1DAI | 2DAI | 3DAI | 7DAI | Total |
| A | J 11_control Vs J 11_infected | 55 | 114 | 15 | 7 | 191 |
| B | J 11_control Vs J 11_infected | 65 | 126 | 19 | 12 | 222 |
| A | JL24_control Vs JL24_infected | 108 | 69 | 66 | 92 | 335 |
| B | JL24_control Vs JL24_infected | 106 | 78 | 55 | 110 | 349 |
| A | JL 24_infected Vs J 11_infected | 70 | 37 | 27 | 5 | 139 |
| B | JL 24_infected Vs J 11_infected | 85 | 78 | 46 | 7 | 216 |
| A | J11_control Vs JL24_control | 182 | 97 | 161 | 45 | 485 |
| B | J11_control Vs JL24_control | 69 | 94 | 78 | 29 | 270 |
|  | Total (daywise) | 740 | 693 | 467 | 307 | 2207 |
|  | Grand total (daywise) | 1283 | 960 | 1061 | 1141 | 4445 |

*Number of induced and repressed genes at different stages of infection like 1^st^ Day after inoculation (1DAI), 2DAI, 3 DAI and 7DAI

**Supplementary Table S3** Expression values of all differentially expressed genes for resistance to in-vtro seed colonization by *Aspergillus flavus* on groundnut (as separate excel document/dataset)

**Supplementary Table S4**. Pathway analysis of differentially expressed genes showing non-redundant pathways that are affected during in-vitro seed colonization of *Aspergillus flavus* in groundnut (as separate excel document/dataset)

**Supplementary Table S5** Effect of 168 sequence variants (SNPs) from differentially expressed genes during *in-vitro* seed colonization of *Aspergillus flavus* in groundnut (as separate excel document/dataset)

**Supplementary Table S6** Allele specific primers designed for 73 differentially expressed genes during *in-vitro* seed colonization of *Aspergillus flavus* in groundnut (as separate excel document/dataset)

**Supplementary Table S7** Number of differentially expressed genes in *Aspergillus* *flavus* (fungus) during pathogenesis from mixed transcriptome analysis

| Aradu_Af_induced | 1DAI | 2DAI | 3DAI | 7DAI |
| --- | --- | --- | --- | --- |
| J 11-Control Vs J 11-Infected | 7 | 12 | 6 | 7 |
| JL 24-Control Vs JL 24-Infected | 8 | 13 | 16 | 6 |
| J 11-Infected Vs JL 24-Infected | 107 | 70 | 113 | 33 |
| J 11-Control Vs JL 24-Control | 0 | 0 | 9 | 0 |
| Aradu_Af_repressed | 1DAI | 2DAI | 3DAI | 7DAI |
| J 11-Control Vs J 11-Infected | 0 | 0 | 0 | 0 |
| JL 24-Control Vs JL 24-Infected | 0 | 0 | 0 | 0 |
| J 11-Infected Vs JL 24-Infected | 18 | 120 | 29 | 14 |
| J 11-Control Vs JL 24-Control | 0 | 0 | 0 | 0 |
| Araip_Af_induced | 1DAI | 2DAI | 3DAI | 7DAI |
| J 11-Control Vs J 11-Infected | 8 | 12 | 6 | 7 |
| JL 24-Control Vs JL 24-Infected | 8 | 11 | 15 | 6 |
| J 11-Infected Vs JL 24-Infected | 107 | 73 | 113 | 42 |
| J 11-Control Vs JL 24-Control | 0 | 0 | 9 | 0 |
| Araip_Af_repressed | 1DAI | 2DAI | 3DAI | 7DAI |
| J 11-Control Vs J 11-Infected | 0 | 0 | 0 | 0 |
| JL 24-Control Vs JL 24-Infected | 0 | 0 | 0 | 0 |
| J 11-Infected Vs JL 24-Infected | 17 | 125 | 29 | 14 |
| J 11-Control Vs JL 24-Control | 0 | 0 | 0 | 0 |

**Supplementary Table S8 List of primers used for qRT-PCR studies**

| S No. | Name | Forward primer (5'-3') | Reverse primer (5'-3') |
| --- | --- | --- | --- |
| 1 | pathogenesis-related protein class partial | AGCCTCATCTATTGCCTCCA | CGATTCCATCACCCCTAAGA |
| 2 | resveratrol synthase | CAGCGCATTTGTGAGAGAAC | CAACGACGGTGCTTTGTATG |
| 3 | cationic peroxidase | TTTCACCATCAGTGCCAGTC | TTTGGGCCTAAGAGGCTTTC |
| 4 | class ii chitinase | CATCACCGGTTGTTCCAAAG | GAACCATAATCACTCAGCCTCTC |
| 5 | heat shock protein 83-like | AGGCGATGTTGAGGAAGTTG | TGGTTATCTCTTCCGGCTTG |
| 6 | microsomal omega-3 fatty acid desaturase | TACTGGCTTTGCTGCTTCTG | TACCGTGGAAAGGAATGGAG |
| 7 | chalcone reductase | AAGGAGGCTATTCAGCTTGG | GAGTTTCGAGCGATTTACGG |
| 8 | ethylene-responsive transcription factor erf060-like | TATCGGATTCTGGCGAAGAC | TGGTTGTGCTGAGTTTGAGG |
| 9 | isoflavone reductase | TGGGCTAGTGTTAAAGCAGGTC | TAACGCCGGATTTCTGGTAG |
| 10 | Conglutin-7 (2S protein 1) (Seed storage protein SSP1) (Seed storage protein SSP2) (allergen Ara h 2) | TCGTCACGTTGGATCTTCTG | AATGGCCAAGCTCACCATAC |
| 11 | lipoxygenase (Phaseolus vulgaris) | AATGGTCCAGGCAAGAATGA | TGTGCTCCATCCTATCCACA |
| 12 | Subtilisin-like protease (EC 3.4.21.-) (Cucumisin-like serine protease) | GGAGTTCGGTTGATGCTGTT | GTGGCTTCCATCACTCCACT |
| 13 | Kunitz-type trypsin inhibitor-like 2 protein (Protease inhibitor from pea 2) | CGTTGTTGAGATCGGTGTGT | CCATCCAAGTTGACGATTCC |
| 14 | Desiccation protectant protein Lea14 homolog | TATGTCCCAATCAGCACCAA | GGGAGATAGCAACAGGGAGA |
| 15 | Seed linoleate 9S-lipoxygenase-2 (EC 1.13.11.58) (Lipoxygenase-2) | GGGCAGAGAGACAATCCAAA | AGGCATATGAGCAGGTCCAA |
| 16 | Alcohol dehydrogenase 3 | GCTTCAAGAGCAGGTCACAAGT | GAGACATCCTCCTTCGTGCATA |
